# Meningitis and Brain Abscess Presenting with Epistaxis in a Woman with Prior Head and Neck Cancer

**DOI:** 10.1155/2015/460208

**Published:** 2015-03-26

**Authors:** Danielle Cross, Rebecca Jeanmonod

**Affiliations:** St. Luke's University Health Network, 801 Ostrum Street, Bethlehem, PA 18015, USA

## Abstract

It is estimated that more than 60% of people have epistaxis in their lifetimes, and as such it is a common complaint encountered in emergency medicine. Although epistaxis is usually self-limited and benign, it can occasionally be a sign of serious underlying pathology. We report a case of epistaxis secondary to invasive squamous cell cancer, ultimately leading to pneumocephalus and brain abscess. We recommend a low threshold for neuroimaging in patients with known prior head and neck cancers presenting with epistaxis, as even resolved epistaxis may be related to serious pathology.

## 1. Case

A 55-year-old woman presented to the emergency department (ED) with epistaxis. She had had unilateral bleeding for about 30 minutes prior to arrival, but the bleeding had resolved by the time of evaluation, and the patient had no other complaints. Her past medical history was remarkable for sinus inverted papilloma and squamous cell cancer several years ago which had been treated with resection and ongoing chemotherapy and proton beam irradiation. On exam, the patient had normal vital signs. Her head and neck exam demonstrated dried blood at her right nare, with no active bleeding or obvious source identified. The patient was subsequently discharged home.

The patient returned to the ED one day later with rapid onset of severe headache with associated nausea and vomiting. She also complained of chills but denied any recent cough, rhinorrhea, ear pain, sore throat, chest pain, or shortness of breath. She denied any measured fevers at home. On exam, the patient was febrile with a temperature of 100.8F and tachycardic at 105. She appeared uncomfortable and was retching. Her HEENT exam as remarkable for chronic facial deformity related to prior tumor resection, including partial removal of frontal bone and reconstructive surgery of the upper nose. The patient had tenderness to palpation of her forehead and frontal scalp, with erythema and crepitation noted. The remainder of her exam was normal.

**Figure 1:**
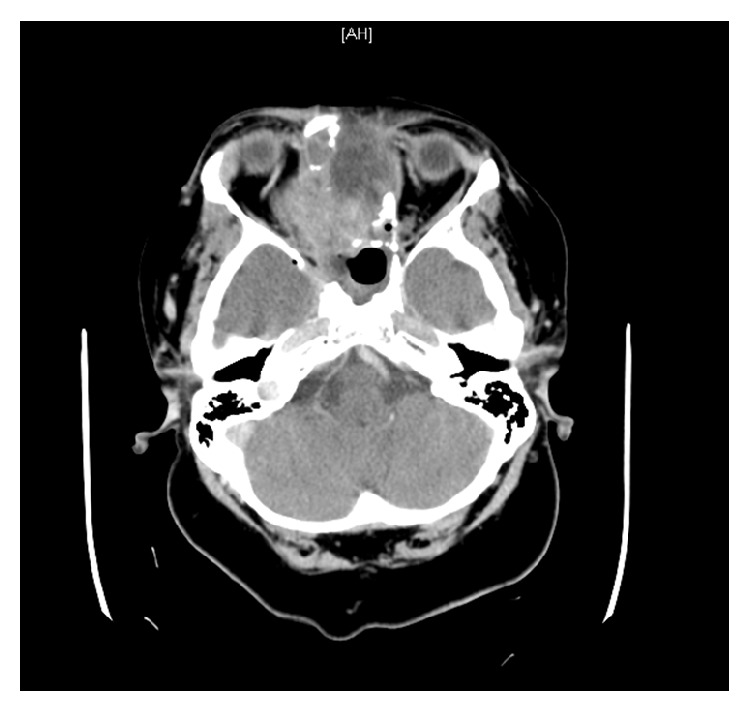
Noncontrasted CT demonstrating tumor extension.

**Figure 2:**
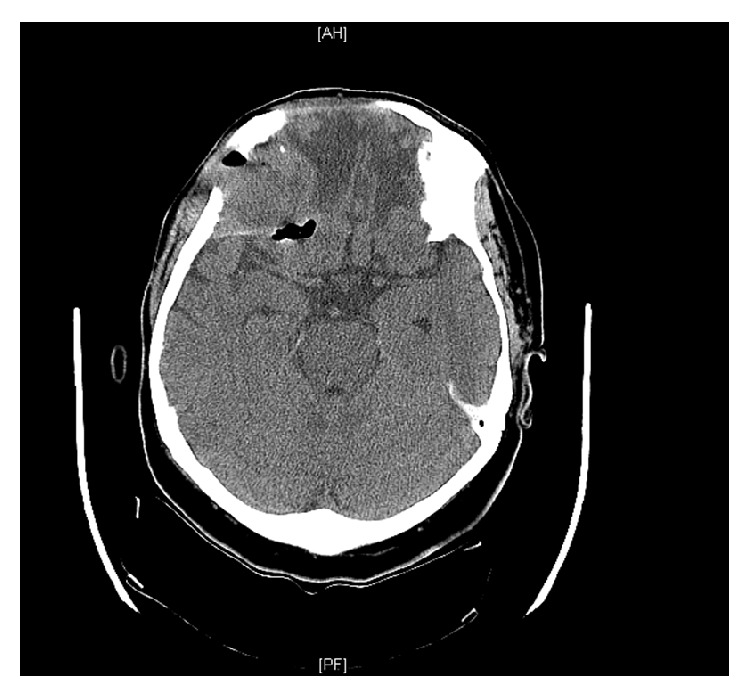
Noncontrasted CT demonstrating brain abscess and pneumocephalus.

The patient's laboratory evaluation was remarkable for a white blood cell count of 13.3, with 91% neutrophils. The remainder of her labs were unremarkable. A computed tomography scan of her head was done, which demonstrated tumor recurrence with intracranial extension and brain abscess (Figures 1 and 2).

The patient was admitted with empiric antibiotics including vancomycin, cefepime, clindamycin, and metronidazole. Her blood cultures grew out Group B Beta hemolytic* Streptococcus*. She underwent resection of the recurrent intracranial tumor and debridement of the infected tissue. She eventually improved and was discharged home three weeks later. She continues to receive proton beam radiation and chemotherapy and is doing relatively well 6 months later.

## 2. Discussion

Epistaxis occurs in an estimated 60% of the population [1–3]. Typically, the bleeding occurs from an anterior source and resolves spontaneously or with direct pressure, cautery, or packing [1–3]. Current guidelines for investigation of the underlying source of bleeding recommend laboratory evaluation for patients at risk of coagulopathy (those on anticoagulation, with liver disease, or family history of bleeding diathesis) and for atypical cases (for instance, epistaxis in a neonate) [4–7], but guidelines on imaging in atraumatic epistaxis are scant.

Sinonasal malignancies are rare, accounting for 1% of all malignancies, and are more common in males over the age of 50 and those of Asian descent [8, 9]. These malignancies typically present with nasal congestion and epistaxis [10], and symptoms are often unilateral and recurrent [2]. Because the symptom constellation overlaps considerably with more benign conditions, these cancers are frequently diagnosed late, and advanced disease at diagnosis is the norm.

Sinonasal malignancies are typically treated with surgical resection and radiation therapy, with many individuals receiving chemotherapy in addition. This radiation therapy places these patients at increased risk of stenosis, aneurysm, and pseudoaneurysm formation within the cranial blood vessels [11, 12]. These vascular insults predispose to cranial vessel rupture and may present with epistaxis which is often massive [11, 13]. In addition to the dramatic epistaxis seen with pseudoaneurysm rupture, patients with sinonasal malignancies will often have epistaxis on tumor recurrence. One study reported epistaxis as the most common clinical manifestation of recurrent disease, present in 38% of their cohort [14].

## 3. Conclusion

Given that patients with known head and neck tumors are at risk of tumor recurrence and intracranial invasion as well as of vascular abnormalities, these patients should undergo CT imaging of the head when presenting to the ED with epistaxis, even with minor presentations. Additionally, in cases of recurrent unilateral epistaxis, consideration should be given to the possibility of nasosinus malignancy, and the patient should be appropriately imaged or, preferably, referred to an otolaryngologist for further evaluation.
